# Valence-induced jumps in coacervate properties

**DOI:** 10.1126/sciadv.abm4783

**Published:** 2022-05-18

**Authors:** Mo Yang, Zachary A. Digby, Yuhui Chen, Joseph B. Schlenoff

**Affiliations:** Department of Chemistry and Biochemistry, Florida State University, Tallahassee, FL 32306, USA.

## Abstract

Spontaneous phase separation, or coacervation, of oppositely charged macromolecules is a powerful and ubiquitous mechanism for the assembly of natural and synthetic materials. Two critical triggering phenomena in coacervation science and technology are highlighted here. The first is the transition from one (mixed) to two (separated) phases of polyelectrolytes coacervated with small molecules upon the addition of one or two charges per molecule. The second is a large jump in coacervate modulus and viscosity mediated by the addition of just one additional charge to a three-charged system. This previously unknown viscoelastic transition is relevant to those aspects of disease states that are characterized by abnormal mechanical properties and irreversible assembly.

## Teaser

The modulus of coacervates made from charged (bio)polymers and small molecules jumps when valency increases from three to four.

## INTRODUCTION

The term “coacervation” was coined by Bungenberg de Jong and Kruyt (*1*) to describe the spontaneous separation of a homogeneous liquid mixture of biopolymers into two or more distinct phases. This type of liquid-liquid phase separation (LLPS) was soon proposed to be one of the mechanisms used to organize and compartmentalize living systems without requiring cell membranes (*2*, *3*). Interest in membraneless organelles has intensified with the discovery of an increasing number of functional droplets within cells and the participation of intrinsically disordered proteins in their formation (*4*–*6*). The nucleolus, an early example of a membraneless organelle, is now known to comprise RNA and proteins with intrinsically disordered regions that undergo LLPS in vitro (*7*).

A focus on biological coacervation is paralleled by extensive research in the basic physical chemistry and materials science of the products of LLPS (*8*–*11*). Unfortunately, research on biological and synthetic coacervates (*12*–*17*) has followed largely separate tracks, although the underlying science is similar. Solid-like products, more common for synthetic systems, are often termed “complexes” (*15*, *16*, *18*). For these, it is possible to access the liquid state by doping with salt or changing other physical variables such as pH (*19*, *20*). Potential driving forces for coacervation/complexation include a number of physical interactions such as charge pairing, or “electrostatics,” between oppositely charged units (*21*). Charge pairing, coupled to and weakened by salt counterions, is driven by the entropic release of counterions (*22*). Enthalpic contributions from hydrogen bonding and hydrophobic interactions (*23*) manifest themselves as upper or lower critical solution temperatures (*24*). Some of the strongest interactions involve arginine, capable of both hydrogen bonding and charge pairing (*25*).

Multivalent interactions lead to net free energies that scale with the number of interacting groups (*26*–*28*). Coacervation in the synthetic and biorealms is typically demonstrated by association between macromolecules, notably RNA as the negative binding partner. However, Mann and co-workers (*29*) and Keating and co-workers (*30*–*33*) have demonstrated the potential of biotically relevant small molecules having few charges to promote LLPS.

**Fig. 1.**
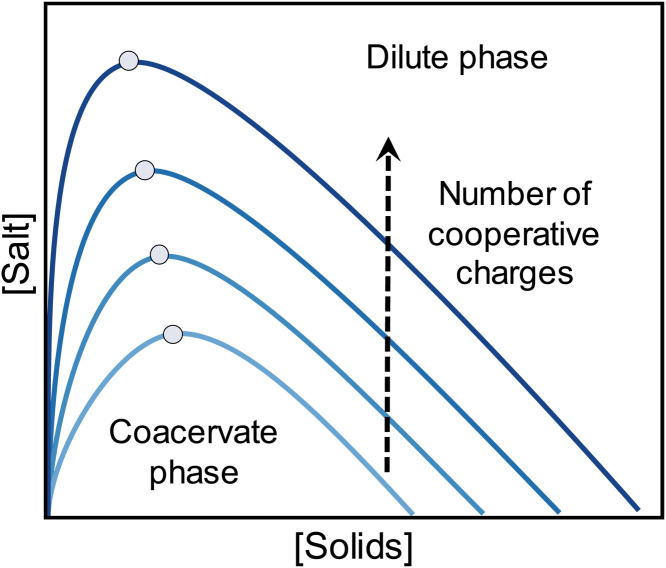
Phase diagram of polyelectrolyte coacervation. No LLPS occurs above the CSCs, shown by the dots, which depend on the molecular weight (or the number of charges per molecule). A larger number of charges stabilizes the coacervate against salt.

Coacervate formation is summarized by phase diagrams, such as those presented in Fig. 1. The boundaries between phases depend principally on salt concentration, type of interacting charges (*34*), number of charges, and charge density. For a specific pair of coacervating polymers, added salt switches off LLPS at the so-called critical salt concentration (CSC) (*35*). The greater the number of charges, the higher the CSC, as illustrated for polymer/polymer coacervates experimentally (*12*, *36*) and by theory (*37*, *38*). Because the CSC is typically near the apex of the phase diagram, it is often shown at the apex, but this is not necessarily the location of the CSC (*39*, *40*). The sensitivity of the CSC to the number of charges increases strongly as the charge density decreases to a few per molecule. In Fig. 1, coacervation in a system just above the CSC may be triggered by the addition of even one charge pair (*31*).

Far more is known about conditions for triggering coacervated droplets than is known about their properties, yet much prior research has highlighted the perceived importance of, for example, viscoelasticity on function (or dysfunction). Many disease states, including neurodegenerative conditions, are characterized by increases in modulus or viscosity, which enable irreversible aggregation and distinct morphology changes (*24*, *41*–*43*). Detailed measurements of materials properties (*44*) such as changes in viscosity (*25*, *45*) have recently come to the forefront in attempts to understand the fundamentals of coacervation.

The present work examines two aspects of the “jumps” in the nature of coacervation. One component was macromolecular, while the other remained small to further illustrate that coacervation requires only one polymeric species. The small components were selected to illustrate the effect of an increasing number of charge-pairing interactions on the formation and properties of polyelectrolyte coacervates (PECs). First, using either a synthetic system with increasing numbers of aromatic sulfonates or a biologically relevant system with increasing numbers of phosphate groups and a polypeptide, a substantial jump in modulus and viscosity occurs for an increase from three to four charges per molecule, providing materials with solid, even glassy, properties. Second, using the phosphate/polypeptide PEC, the phase boundary in physiological salt concentration between unassociated and associated molecules is crossed with the addition of two charges by in situ hydrolysis.

## RESULTS AND DISCUSSION

**Fig. 2.**
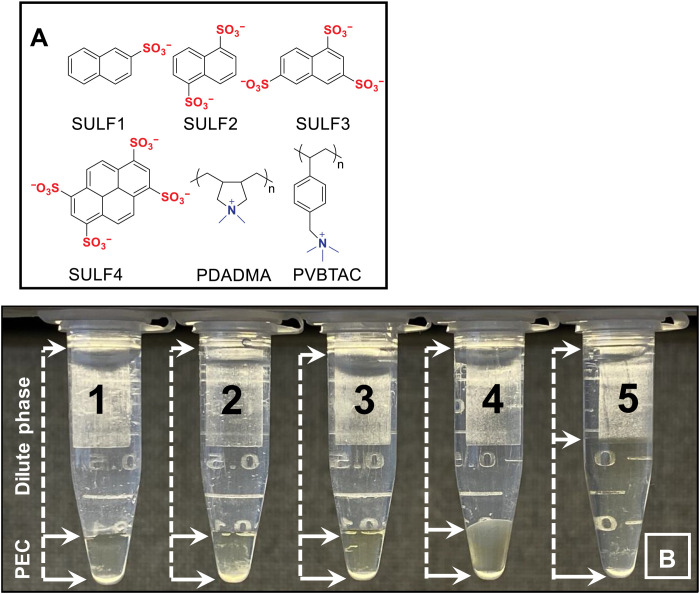
Molecules used for SULF series of PECs. (**A**) Small and macromolecular-charged partners used in this study. SULF1, 2-naphthalenesulfonic acid sodium salt; SULF2, 1,5-naphthalenedisulfonic acid disodium salt; SULF3, 1,3,6-naphthalenetrisulfonic acid trisodium salt; SULF4, 1,3,6,8-pyrenetetrasulfonic acid tetrasodium salt; PDADMA, poly(diallyldimethylammonium); PVBTAC, poly(vinylbenzyl trimethylammonium chloride). (**B**) Images of (1) SULF1/PDADMA, (2) SULF2/PDADMA, (3) SULF3/PDADMA, (4) SULF4/PDADMA, and (5) SCN/PDADMA coacervates (lower phase) in 1.5-ml centrifuge tubes. The upper phase is termed the dilute phase (indicated by the arrows).

We prepared the first series of coacervates using the sulfonate/quaternary ammonium charge pair. A set of molecules bearing from one to four sulfonates each is shown in Fig. 2A. We complexed these with the polycation poly(diallyldimethylammonium) (PDADMA) fractionated to provide a relatively narrow distribution of molecular weights (see fig. S1). Coacervate droplets were centrifuged to yield a continuous polymer-rich phase and a “dilute” phase (see Fig. 2B). While properties and composition are known to change with salt concentration (*46*), in the current study, we maintained [NaCl] near 0 or 0.15 M.

All compositions were close to stoichiometric in terms of SO_3_^−^:PDADMA^+^ charge ratios (Table 1 and fig. S2). The water and solids content, determined by drying (Table 1), showed only a slight change in composition across the sulfonate series. The critical concentration of NaCl required to revert the two-phase system back into one phase increased substantially with the number of sulfonates. Thus, only the 1,3,6,8-pyrenetetrasulfonic acid tetrasodium salt hydrate (SULF4)/PDADMA coacervate would form at [NaCl] > 0.53 M.

**Table 1.** Stoichiometry, polyelectrolyte volume fraction, and CSC of SULF/PDADMA PECs, as well as heat of complexation determined by isothermal titration calorimetry. PE, polyelectrolyte.

**Sample**	**Stoichiometry** **(SO_3_^−^:PDADMA^+^)**	**% PE volume** **fraction**	**CSC (M)**	**Total charges per** **SULF molecule**	**Δ*H* per SULF molecule** **±100 (J mol^−1^)**	**Δ*H* per SO_3_^−^ group** **±100 (J mol^−1^)**
SULF1/PDADMA	0.97:1.00	20.4	0.04	1	−950	−950
SULF2/PDADMA	1.00:1.00	20.1	0.08	2	−5320	−2660
SULF3/PDADMA	0.99:1.00	22.8	0.53	3	−7790	−2600
SULF4/PDADMA	0.99:1.00	28.6	2.68	4	−23,500	−5880

While entropy provides a driving force for complex formation due to the liberation of counterions on the polymer, much of the driving force in the SULF/PDADMA series comes from the enthalpy of complexation, which becomes more exothermic with the number of ─SO_3_^−^ per molecule and illustrates the influence of binding polyvalency or cooperativity (Table 1). There is a jump in the enthalpy per SO_3_^−^ going from 1,3,6-naphthalenetrisulfonic acid trisodium salt hydrate (SULF3) to SULF4, probably a result of the added aromaticity/hydrophobicity.

**Fig. 3.**
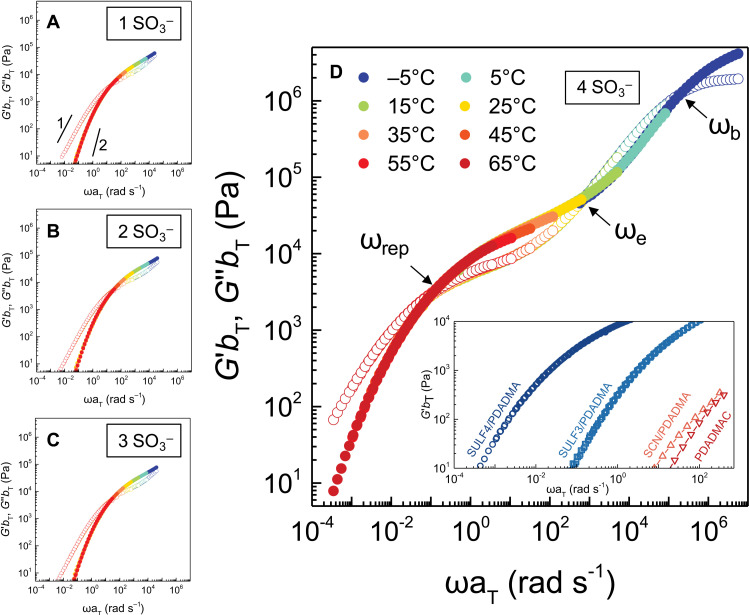
VR for SULF PECs. (**A**) SULF1/PDADMA, (**B**) SULF2/PDADMA, (**C**) SULF3/PDADMA, and (**D**) SULF4/PDADMA. Storage modulus *G*′ (filled symbols) and loss modulus *G*″ (open symbols) are shown as a function of frequency, shifted from different temperatures according to time-temperature superposition with a reference temperature of 25°C. Inset compares *G*′ in the terminal regime for SULF4/PDADMA, SULF3/PDADMA, SCN/PDADMA, and 27.5 wt % of PDADMAC solution. Characteristic relaxation rates ω_rep_, ω_e_, and ω_b_ are shown in (D).

**Fig. 4.**
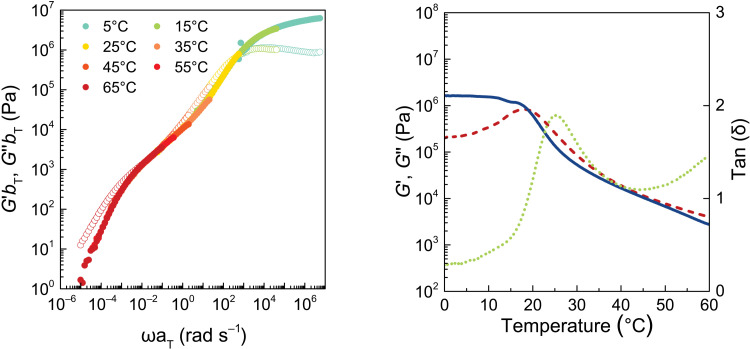
VR for SULF4/PVBTA. (**Left**) Frequency response. (**Right**) Temperature response showing a glass transition at about 25°C; solid line, *G*′; dashed line, *G*″; dotted line, tanδ. See fig. S5 for shift factors.

Because all hydrated coacervate phases were macroscopic, large-scale rheological measurements could be recorded to determine the viscoelastic response (VR) as a function of frequency (storage modulus *G*′ and loss modulus *G*″ in Fig. 3). Figure S3 shows viscosity and shift factors used to perform time-temperature superposition (*47*). There were relatively minor differences between the VR of coacervates made with 2-naphthalenesulfonic acid sodium salt (SULF1) through SULF3 (Fig. 3, A to C). SULF4/PDADMA exhibited a remarkable jump in modulus and a full range of frequency-dependent (Fig. 3D and fig. S3) and temperature-dependent (fig. S4) VR from liquid-like to rubbery to almost glassy. The (zero shear) viscosity remains virtually constant for SULF1 to SULF3 and then jumps by a factor of 160 for SULF4 (Table 2). Very little separates the SULF series of PECs in terms of composition. The only clue to the extraordinary properties of SULF4/PDADMA provided in Fig. 2 is the skewed interface at the bottom of the centrifuge tube. The unusually glassy nature of a coacervate formed from a polyelectrolyte and a small molecule with just four charges is emphasized by the observation of a glass transition temperature, *T*_g_, at around room temperature for a coacervate between SULF4 and the strongly pairing polycation (*34*) poly(vinylbenzyltrimethyl ammonium) (PVBTA) (Fig. 4). We did not observe *T*_g_’s for the other systems, which we assumed to be below 0°C.

**Table 2.** Dynamic properties of SULF/PDADMA PECs.

**Sample**	**Zero shear viscosity, η_o_** **±100 (Pa s)**	**Plateau modulus, *G*_o_ (Pa)**	**Reptation rate, ω_rep_ (s^−1^)**	**Entanglement rate, ω_e_ (s^−1^)**
SULF1/PDADMA	1400	25,000	22	60,000
SULF2/PDADMA	1300	32,000	28	70,000
SULF3/PDADMA	1300	33,000	25	60,000
SULF4/PDADMA	210,000	25,000	0.1	890

**Fig. 5.**
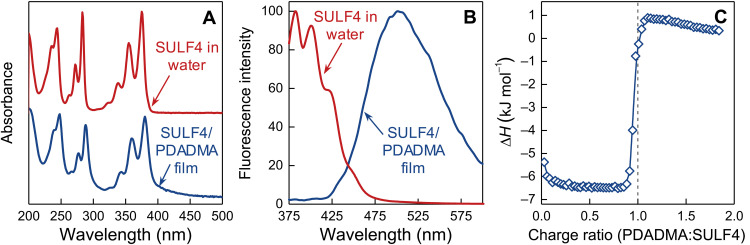
Absorption, emission, and calorimetry of SULF4/PDADMA. (**A**) UV-vis absorption spectra of SULF4 in water and SULF4/PDADMA film. (**B**) Fluorescence emission spectra of SULF4 in water and SULF4/PDADMA film. (**C**) Isothermal calorimetry for the coacervation of PDADMAC (10 mM) injected into SULF4 (0.25 mM) in 0.05 M NaCl at 25°C.

The ultraviolet-visible (UV-vis) absorption and emission spectra of SULF4/PDADMA shown in Fig. 5 (A and B) yield insight on a possible mechanism for the jump in association enthalpy (an example of the calorimetry is shown in Fig. 5C). While the UV-vis absorption spectra of SULF4 in water and in the PEC are similar, there is a strong red shift of the emission maximum in the latter, indicating excimer formation due to stacking of the planar SULF4 molecule (*48*).

Strong differences in viscoelastic properties are also illustrated in Fig. 3D, which compares *G*′ for SULF3/PDADMA and SULF4/PDADMA with a solution of the chloride salt of PDADMA by itself at a similar weight % (wt %) to that found in the SULF coacervates. Figure 3D includes the VR of a coacervate between thiocyanide (SCN^−^), a monovalent ion on the chaotropic end of the Hofmeister series, and PDADMA (see Fig. 2B for an image of this LLPS). The SCN/PDADMA coacervate contains more water (Fig. 2B) and is much less viscous than its single-charged SO_3_^−^ counterpart, SULF1. It should be stressed that coacervation of the appropriate macromolecule may be induced by an ionic species carrying just one charge, drawing attention to the overlap between terminologies used to describe LLPS, including “condensation,” “demixing,” “precipitation,” and “complexation.” Such monovalent species cannot form bridges between macromolecules.

For comparison, the viscoelastic properties of a 27.5 wt % solution of PDADMA(Cl) at a concentration similar to that of the SULF series are shown in Fig. 3D. This experiment was intended to show that the dynamics are slowed much more when SULF is used to prepare coacervates of the polymer having similar weight %. Although experiments are performed without added salt, the viscosities of all coacervates are expected to decrease were NaCl to be added (*13*, *49*).

The shape of the VR in Fig. 3D is characteristic of an entangled polymer (*50*). The rubbery plateau modulus, recorded as the minimum of tanδ (see fig. S3), remained approximately constant for the entire SULF series at about 3 × 10^4^ Pa (Table 2), which is expected if the volume fractions of polymer are comparable. Because the coacervates are stoichiometric, the SO_3_^−^ density and the density of SO_3_^−^/DADMA^+^ charge pairs also remained roughly constant. The characteristic relaxation rates in Fig. 3D, indicated by the crossings of *G*′ and *G*″, include the reptation rate, ω_rep_; estimated entanglement relaxation rate, ω_e_; and, at the highest frequencies, the relaxation rate between the minimum number of monomer units, ω_b_, visible only for SULF4 (~10^5^ s^−1^). (*10*)

### Valence formation threshold and modulus jump with inorganic phosphates

**Fig. 6.**
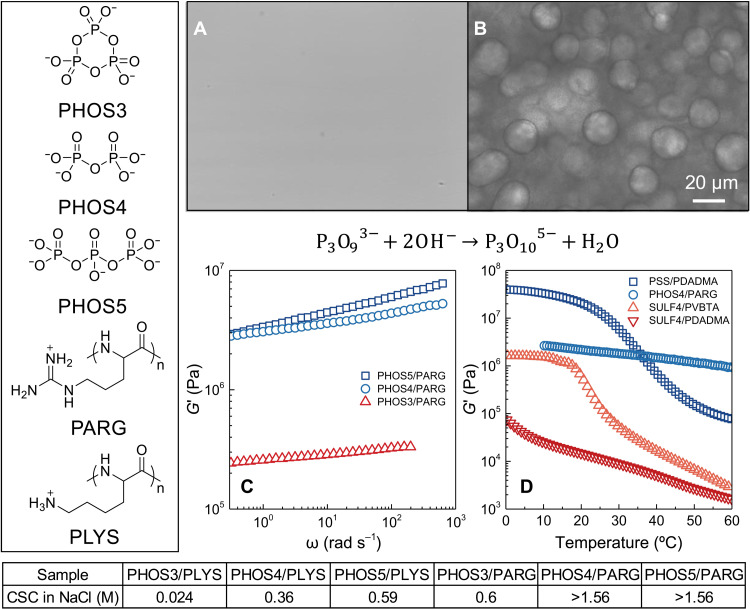
Micrographs and viscoelasticity of PHOS PECs. (**A**) Micrographs of a fresh mixture of polylysine, PLYS, and PHOS3 in 0.15 M NaCl (pH 7) and (**B**) 12 hours after adding NaOH to hydrolyze the PHOS3 to PHOS5. (**C**) *G*′ versus frequency for PHOS/PARG coacervates at 37°C in 0.15 M NaCl (pH 7). See fig. S6 for *G*″ and tanδ. (**D**) *G*′ versus the temperature of PSS/PDADMA, SULF4/PDADMA, SULF4/PVBTA, and PHOS4/PARG in 0.01 M NaCl at 0.1 Hz. Ramp rate = 1°C min^−1^ for PSS/PDADMA, SULF4/PDADMA, and SULF4/PVBTA; ramp rate = 2°C min^−1^ for PHOS4/PARG. The table at the bottom summarizes the CSC of PHOS3/PLYS, PHOS4/PLYS, PHOS5/PLYS, PHOS3/PARG, PHOS4/PARG, and PHOS5/PARG in NaCl. PARG is insoluble at > 1.56 M NaCl.

Aromatic interactions in the SULF/PDADMA series may provide additional hydrophobic or π-π bonding to help assemble coacervates. To provide a completely different, aromatic-free system, we made a more biologically relevant series of coacervates using inorganic phosphates (PHOS) and polyarginine (PARG) (Fig. 6). These systems are comparable to those using nucleoside phosphates, such as adenosine triphosphate, adenosine diphosphate, or adenosine monophosphate, and cationic polypeptides (*29*) or synthetic polycations (*33*). We prepared coacervates at physiological NaCl concentrations and recorded the VR. Figure 6C shows that there is a strong increase in modulus from trisodium trimetaphosphate (PHOS3) to sodium pyrophosphate tetrabasic (PHOS4), with a minor increase to sodium triphosphate pentabasic (PHOS5).

This system again exhibits a step in VR properties going from three to four charges. The accessibility to the charges on PHOS is not a limitation here. Using ChemDraw 20, we estimated the volume and solvent-accessible area of PHOS3 to be 112 Å^3^ and 304 Å^2^, respectively, while those of PHOS4 were 85 Å^3^ and 263 Å^2^. Thus, Arg or Lys repeat units should be able to engage the charges on PHOS3.

To demonstrate the importance of the functional group in determining coacervation, no LLPS resulted when mixing polylysine instead of PARG with PHOS3 in 0.15 M NaCl. Base-induced hydrolysis (ring opening) of PHOS3 to PHOS5 yielded coacervate droplets from this mixture (Fig. 6B). PHOS5/poly-l-lysine hydrochloride (PLYS) coacervation under the conditions used (pH 7, 0.15 M NaCl) is triggered by an increase in valency of the inorganic phosphate from three to five, which increases the CSC from 0.024 to 0.59 M (Fig. 6 compares CSCs for the PHOS series).

The VR of polymers with “sticky” interactions is known to be controlled by the lifetime of these interactions, i.e., charge pairs in the present case (*51*). The fastest relaxation rate [using the classical terminology of polymer physics (*50*)], from the smallest group of paired charges (*10*), is given by ω_b_, which was only observable for SULF4/PDADMA (Fig. 3D). A lower ω_b_ shifts all characteristic relaxation rates to lower frequencies. This direct connection between charge pair lifetimes and macromolecule dynamics leads to higher viscosities and moduli. PARG forms more strongly associating charge pairs (implying longer lifetimes), illustrated by a higher CSC (see table in Fig. 6) with PHOS than does PLYS, imparting greater viscosity and modulus to the coacervate (see fig. S7 for a comparison of PHOS5/PARG and PHOS5/PLYS).

### Possible mechanism for jump in modulus

The mechanism for the jump in coacervate VR induced by a small-molecule valency change from three to four is of great interest. Multivalency influences relaxation rate to some extent. For comparison, the VR of a polymeric sulfonate (PSS) coacervated with PDADMA is also shown in Fig. 6D and fig. S8. The VR of this and other combinations of synthetic polyelectrolytes have been intensely studied. Extensive, random charge pairing between two macromolecules results in LLPS and liquid-like or solid-like materials (*10*, *11*, *13*, *52*). In a couple of examples, the actual rate of charge unpairing (the inverse of the pair lifetime) has been inferred from ion conductivity measurements (*10*, *53*).

The jump in modulus for the SULF3 to SULF4 is seen in Fig. 3 by a shift of VR to lower frequencies. Both ω_rep_ (Table 2) and the terminal region of *G*′ (inset of Fig. 3D) shift by a factor of about 300; all other parameters remain equal (e.g., volume fraction of polymer and length of polymer chain). A small ion with three charges can engage one polymer chain with two charge pairs and another with one charge pair. Chain relaxation is thus as fast as the breaking of the “weak link:” a single pair of charges. Four-valent ions may bridge two polymer chains with two charge pairs on each. Chain relaxation now relies on breaking two charge pairs simultaneously, which is much less likely and much slower (*10*). Therefore, all chain dynamics are slowed and the viscosity increases substantially.

To estimate the change in the number of interactions involved at ω_b_, we replotted the highest temperature points from the frequency shift data for SULF3/PDADMA and SULF4/PDADMA (fig. S3) as an Arrhenius plot in fig. S9. At sufficiently high temperatures, polymer dynamics are known to exhibit Arrhenius behavior (*10*, *50*). The activation energy for SULF3/PDADMA was 57 kJ mol^−1^ [about the same as those for SULF1 and 1,5-naphthalenedisulfonic acid disodium salt (SULF2)/PDADMA], whereas that for SULF4/PDADMA was 99 kJ mol^−1^, almost twice as much, consistent with a doubling of the SULF-PDADMA interactions at ω_b_ limiting dynamics. At present, we do not know whether this number doubles from one to two or from two to four going from SULF3 to SULF4.

### Physical and biological relevance

Bungenberg de Jong (*1*) initially thought that coacervates were aggregates of colloidal particles. Two decades later, he distanced himself from this view and took the modern perspective that coacervates are homogeneous phases (*2*). His categorization of coacervates as “simple,” relying on nonionized groups for interaction, and “complex,” which are driven by charges and the formation of “salt bonds” (charge pairs), was probably too basic for biomolecule coacervation but adequate for synthetic systems that can be designed with less complexity. He relied heavily on hexol (*2*), Co[(μ-OH)_2_Co(NH_3_)_4_]_3_(NO_3_)_6_, a hexavalent cobalt complex first made by Jörgensen (*54*), and later studied by Werner (*55*), to coacervate acidic proteins. As seen in Fig. 2, coacervation of a synthetic polyelectrolyte relying on charge needs only a single-charged hydrophobic ion to induce phase separation. The “strength” of coacervation, reported by the CSC, cannot be judged solely by the amount of water expelled or the “hydrophobicity” of the coacervating components (compare the coacervate volumes and CSC in Table 1). Cooperativity of charge pairing also plays a major role in the CSC. This cooperativity is not reflected in the viscoelasticity until four charges are involved. In a recent mechanism for polymer/polymer coacervates, we concluded that the minimum cooperatively rearranging unit in PEC dynamics was an exchange of two Pol^+^Pol^−^ pairs with a relaxation rate for PDADMA/PSS of about 10^4^ s^−1^ (*56*). A ω_b_ of about 10^5^ s^−1^ in Fig. 3D is consistent with pair exchange in a material similar to PDADMA/PSS containing more water.

Biological structures also exhibit a substantial range of VR. The loss of dynamics and physical reversibility at the molecular level may lead to larger-scale aggregation and morphology variations in organs and organelles. Aggregation may be induced by functionality transformation (e.g., lysine to arginine), change of charge density [e.g., (de)phosphorylation], misfolding (allowing two strongly interacting groups, which would normally be held apart, to approach), a change of registry/sequence of opposite charges, or similar changes in chaperones that facilitate disassembly (*57*). The significant finding in our work is that a sudden increase in local cooperativity, leading to a strong increase in solid-like character, occurs with a transition from three to four nearby charge pairs. This mechanism is in addition to the slow aging of tissues from the loss of (plasticizing) water, greater cross-linking, and other aging mechanisms.

## MATERIALS AND METHODS

### Materials

SULF1 (90%), SULF2 (95%), SULF3, SULF4 (98%), PHOS3 (95%), PHOS4 (95%), PHOS5 (98%), NaCl, KBr, sodium azide, and MOPS were from Sigma-Aldrich. Sodium thiocyanate (NaSCN) was from VWR. Medium–molecular weight (MW) PDADMA chloride [PDADMAC; 20 wt % in water; MW, 200,000 to 350,000] was from Sigma-Aldrich. Poly-l-arginine hydrochloride (MW, 38,500) and PLYS (MW, 66,000) were from Alamanda Polymers. Poly(vinylbenzyl trimethylammonium chloride) (PVBTAC; 27 wt % in water; MW, 100,000) was from Scientific Polymer Products. Deionized water (18 megohms·cm) (Barnstead, Nanopure) was used to prepare all solutions.

### PDADMAC fractionation

Commercial samples of PDADMAC usually have broad molecular weight distributions (MWDs), weight-average molecular weight (*M*_w_)/number-average molecular weight (*M*_n_). PDADMAC specified to be in the MW range of 200,000 to 350,000 by Sigma-Aldrich was fractionated to narrow the MWD of PDADMAC from 3.3 to 1.4. As-received PDADMAC solution was diluted to 10 wt % in water. Acetone (99.5%; VWR) was gradually added into 100 ml of 10 wt % PDADMAC solution until the solution became cloudy. Then, the cloudy solution was centrifuged at 6000 rpm until the supernate became transparent. The supernate was collected, and the same fractionation procedure was repeated two more times to remove most of the high–molecular weight PDADMAC. Last, the third fraction of PDADMAC was collected and dried at 120°C for 24 hours.

### Size exclusion chromatography

The *M*_w_, *M*_n_, and MWD of PDADMAC before and after fractionation were determined by size exclusion chromatography with light scattering detection. Fifty microliters of PDADMAC (2 mg ml^−1^) in 0.3 M NaNO_3_ was injected through a 300-mm by 8-mm PSS Inc. Novema Max Lux 1000-Å analytical column guarded by a 10-μm Novema Max Lux guard column. 0.3 M NaNO_3_ preserved with 200 parts per million (ppm) of NaN_3_ was used as the mobile phase. A DAWN-EOS multiangle light scattering detector in series with a rEX refractometer (Wyatt Technology) was used to collect molecular weight data. The refractive index increment, d*n*/d*c*, for PDADMAC in 0.3 M NaNO_3_ was 0.186, which was measured with the refractometer using an offline mode. Figure S1 shows the chromatograms.

### Sulfonate/PDADMA coacervates

SULF1, SULF2, SULF3, and SULF4 were dried under vacuum at 120°C for 24 hours. Ten milliliters of 0.3 M PDADMAC (fractionated) solution was mixed with 10 ml of 0.3 M SULF1, 0.15 M SULF2, 0.1 M SULF3, and 0.075 M SULF4 to form SULF1/PDADMA, SULF2/PDADMA, SULF3/PDADMA, and SULF4/PDADMA coacervates, respectively. The mixtures were centrifuged at 6000 rpm for 24 hours to collect the coacervates.

### SULF4/PDADMA film

Bulk SULF4/PDADMA coacervates were too absorbing to study by UV-vis transmission spectroscopy. Therefore, SULF4/PDADMA films were built on fused silica using a robot (StratoSequence V, NanoStrata Inc.) to perform a layer-by-layer assembly. A “bilayer” was made by dipping the silica in 1 mM PDADMAC solution for 5 min, followed by 5 min of dipping in 10 mM SULF4. After 15 bilayers of SULF4/PDADMA film were deposited, the silica was removed, and films were dried under a stream N_2_.

### Phosphate-polypeptide coacervates

Poly-l-arginine, poly-l-lysine, and phosphate salts were vacuum-dried at room temperature for 24 hours before transfer to an argon-filled glove box to be weighed. 0.125 M poly-l-arginine and poly-l-lysine solutions in 0.15 M NaCl, 20 mM 3-(N-morpholino)propanesulfonic acid (MOPS) buffer, and 200 ppm of sodium azide were mixed with equal volumes of phosphate salt solutions at molar concentrations resulting in stoichiometric charge ratios. The resulting coacervates were then vortexed for 10 min and centrifuged for 4 hours at 12,000 rpm. The supernate was removed from the centrifuge tube, and coacervates were partially dried under vacuum for 6 hours. The coacervates were placed into a stainless steel 8-mm-diameter mold and pressed at room temperature for 24 hours. The PEC tablets were then removed from the mold and placed into a 20-ml vial filled with a solution at 0.15 M NaCl, 20 mM MOPS (pH 7), and 200 ppm of sodium azide to equilibrate for 24 hours.

### Nuclear magnetic resonance spectroscopy

The stoichiometries of SULF/PDADMA coacervates were determined using solution ^1^H nuclear magnetic resonance (NMR) spectroscopy. NMR samples were prepared by dissolving the dry PEC in a solution of KBr in D_2_O. This allowed the number of protons on both the small molecules and polyelectrolytes to be measured. The KBr concentrations used in NMR sample preparation were different from one to another because the CSCs required to fully dissociate the PECs were different. For SULF1/PDADMA, SULF2/PDADMA, and SULF3/PDADMA, 10 mg of the dry PEC was dissolved in 1.0 M KBr in D_2_O, whereas 10 mg of dry SULF4/PDADMA was dissolved in 3.0 M KBr in D_2_O. An AVANCE 600-MHz NMR (Bruker) was used to acquire the spectra. NMR spectra are shown in fig. S2.

### UV-vis spectroscopy

UV-vis absorption spectra were obtained on a UV 2450 absorption spectrophotometer (Shimadzu). The silica slide bearing the SULF4/PDADMA film was mounted on a custom-designed sample holder. The absorption of 10^−5^ M SULF4 in water was also measured in a fused silica cuvette.

### Emission spectroscopy

SULF4/PDADMA film fluorescence emission spectra were recorded on a Horiba Fluoromax-4 spectrofluorometer. Samples were excited at 370 nm, and emission was measured from 375 to 600 nm. Emission intensity was recorded every 1 nm, excitation and emission slit widths were 1 nm, and the integration time was 0.5 s.

### Critical salt concentration

The CSCs of SULF/PDADMA coacervates were determined with the gradual addition of NaCl to a suspension (1 mg ml^−1^) of PEC particles, prepared by mixing salt-free solutions of PEC components. The CSC was taken to be the [NaCl] at which solutions became clear (visibly and by turbidimetry). The CSCs of PHOS/PARG and PHOS/PLYS PECs were also determined using this method. In the case of PHOS4/PARG and PHOS5/PARG, it was found that the PECs were insoluble up to 6 M NaCl, and therefore, the CSC is unknown.

### Isothermal titration calorimetry

PDADMAC was dialyzed (3500 MW cutoff tubing; SnakeSkin, Thermo Fisher Scientific) against deionized water for 2 days, with water replacement every 12 hours. The PDADMAC solution was then freeze-dried (Labcono, FreeZone 105) to a powder. The sulfonate salts (SULF1, SULF2, SULF3, and SULF4) and PDADMAC powder were dried at 110°C for 4 hours and then immediately moved into an argon-filled glove box to be weighed.

Isothermal titration calorimetry (ITC) was performed using a VP-ITC (MicroCal Inc.) calorimeter. The ITC was calibrated with an internal *y*-axis calibration followed by a standard titration between hydrochloric acid and tris base. All samples were degassed for 10 min at room temperature. Approximately 300 μl of a 10 mM PDADMAC solution was loaded into the syringe. Ten microliters of the PDADMAC solution was manually discharged from the syringe to relieve any back pressure from the loading process. Before filling, the sample cell (1.4138 ml) was washed with the SULF solution. To accommodate the amount of charge on one molecule and the limited number of injections that are allowed, the trivalent SULF3 and quadrivalent SULF4 salts were 0.25 mM solutions, while the monovalent and divalent salts were 0.5 mM solutions. The syringe was rotated at 260 rpm in the sample cell with an injection size of 4 μl per aliquot at a rate of 0.50 μl s^−1^, with 240 s between injections. The heat flow was recorded as a function of time at 25.0°C. Enthalpies were calculated by summing the total heat generated to the end point with a correction for the background dilution enthalpy (see fig. S10 for ITC thermograms). The dilution enthalpy was determined from the addition of 10 mM PDADMAC into water under identical conditions.

### Viscoelastic response

Measurements of linear VRs were performed using a strain-controlled DHR-3 rheometer (TA Instruments) with Peltier temperature control. A 20-mm parallel plate was used for all experiments except for polypeptide-PHOS coacervate, where an 8-mm parallel plate was used. A custom-designed lower plate in a solution reservoir with a cap was used to prevent evaporation. The coacervates were first transferred onto the lower plate. The upper plate was then lowered onto the samples to provide a ~100-μm gap. The excess coacervate was trimmed off, and the desired aqueous solution was added to the solution reservoir to maintain the environment for coacervates. Frequency sweep experiments were performed on the samples at temperatures ranging from −5° to 65°C. Fifteen minutes was allowed for samples to reach temperature equilibrium. Temperature ramp experiments were carried out at 1 Hz with a ramp rate of 1°C min^−1^. Strain sweep experiments were performed from 0.01 to 100% strain to ensure that all responses were within the linear viscoelastic regime.

### PHOS/PLYS coacervate hydrolysis

A 0.5-ml solution (pH 7, 0.15 M NaCl, 200 ppm of sodium azide, and 20 mM MOPS) with 0.15 M polylysine was mixed with 0.5 ml of a 0.05 M PHOS3 solution (identical pH, salts, and buffer). No coacervation was observed. The solution was vortexed for 5 min, and the pH was increased to 9 by adding concentrated NaOH. The solution was then placed on a tissue culture plate and moved to the imaging stage of the Nikon Eclipse Ti-DH Inverted Microscope equipped with a Photometrics Coolsnap HQ2 CCD camera (1392 by 1040, 6.45 μm^2^ of pixels). The culture plate was left on the imaging stage for 12 hours without movement while images were taken periodically.
