# Supplementary material for: Valence-induced jumps in coacervate properties
Source: Sci Adv. 2022 May 18;8(20):eabm4783. doi: 10.1126/sciadv.abm4783 (PMC9116606; doi:10.1126/sciadv.abm4783)
Supplement: Supplementary file 1 — Figs. S1 to S10 [file sciadv.abm4783_sm.pdf]

Science Advances
AAAS

Supplementary Materials for

Valence-induced jumps in coacervate properties

Mo Yang, Zachary A. Digby, Yuhui Chen, Joseph B. Schlenoff*

*Corresponding author. Email: jschlenoff@fsu.edu

Published 18 May 2022, *Sci. Adv.* **8**, eabm4783 (2022)
DOI: 10.1126/sciadv.abm4783

This PDF file includes:

Figs. S1 to S10

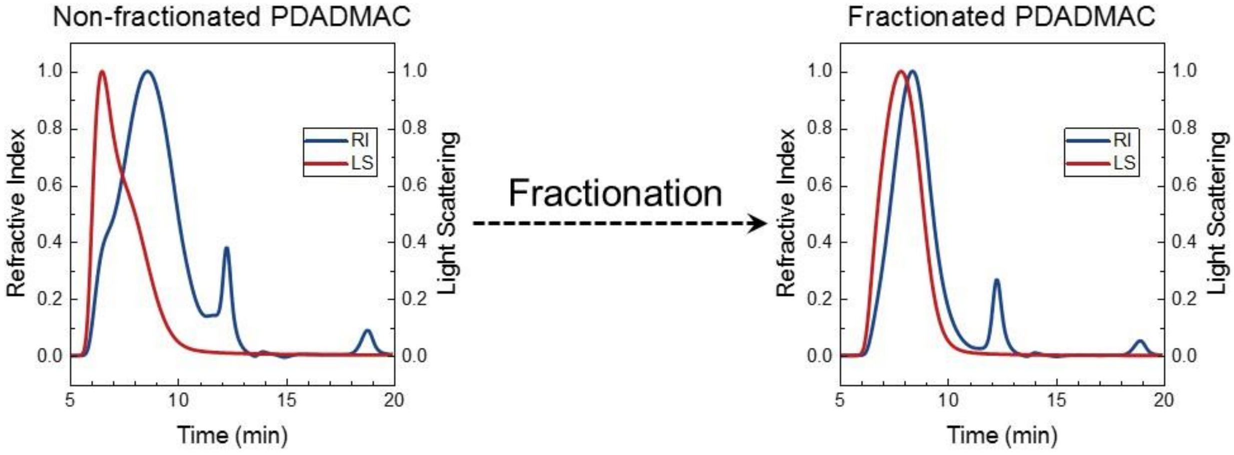

Mn	Mw	PDI
1.54×10^5	5.01×10^6	3.25

Mn	Mw	PDI
2.30×10^5	3.25×10^5	1.42

Fig. S1. **SEC chromatograms of as-received "medium" molecular weight PDADMAC and fractioned PDADMAC used in this study**. Number average molecular weight, M_n, weight average molecular weight, M_w, polydispersity index (PDI = M_w/M_n).

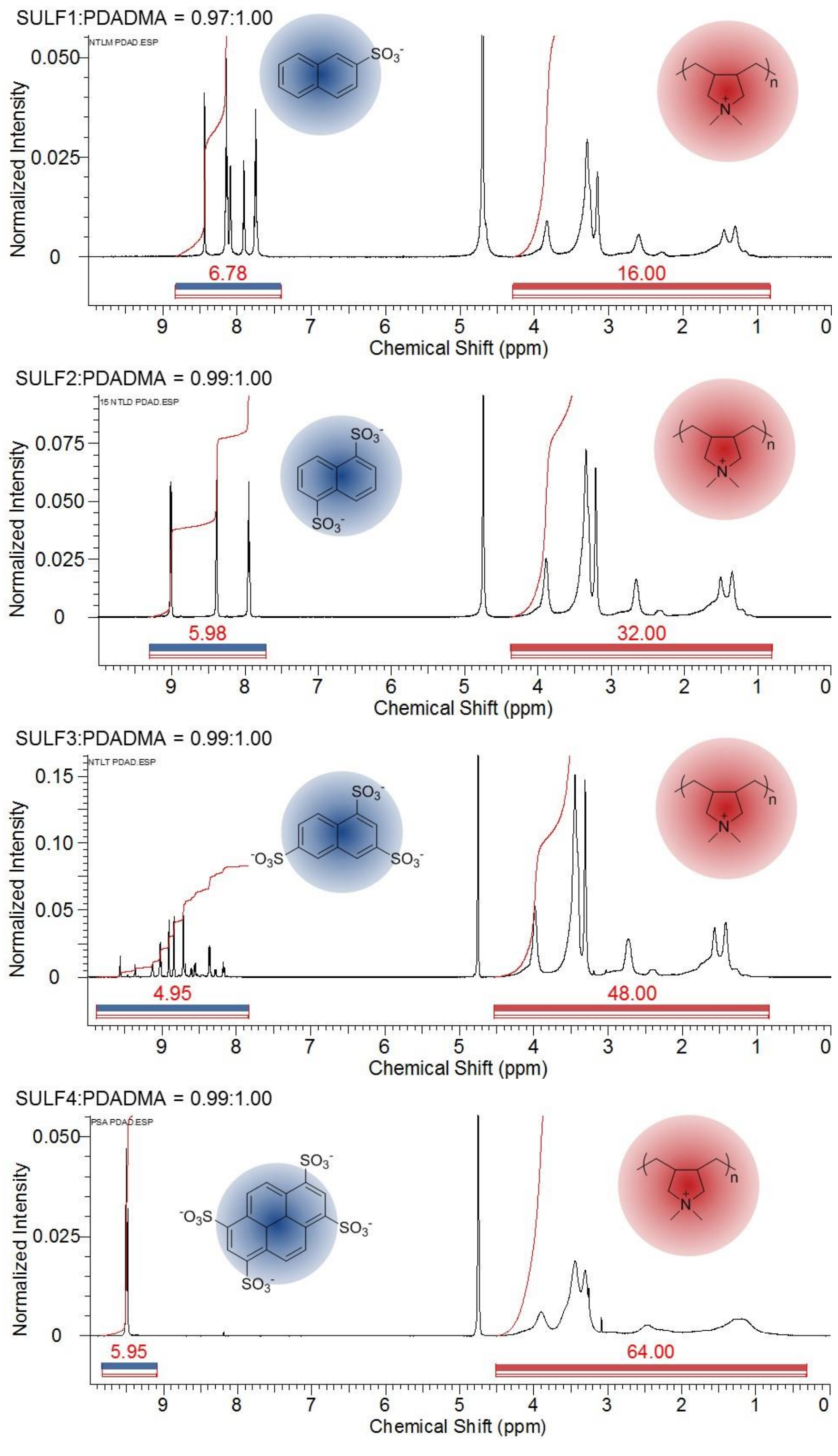

Fig. S2. ¹H NMR spectra of SULF1/PDADMA, SULF2/PDADMA, SULF3/PDADMA and SULF4/PDADMA coacervate dissolved in KBr in D_2O solution at room temperature. Aromatic ^1H >6 ppm. Aliphatic ^1H <5 ppm. Integrated peak areas for these regions are shown.

Time-temperature superposition was achieved using the two equations below:

$$G'(\omega, T) = G'(a_T\omega, T_o)/b_T$$
$$G''(\omega, T) = G''(a_T\omega, T_o)/b_T$$

Where ω is the frequency, T is the experimental temperature, a_T is the x-axis shift factor and b_T is the y-axis shift factor. T_0 is reference temperature, which was 25 °C.

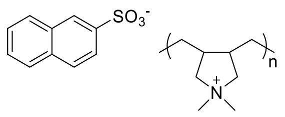

SULF1 PDADMA

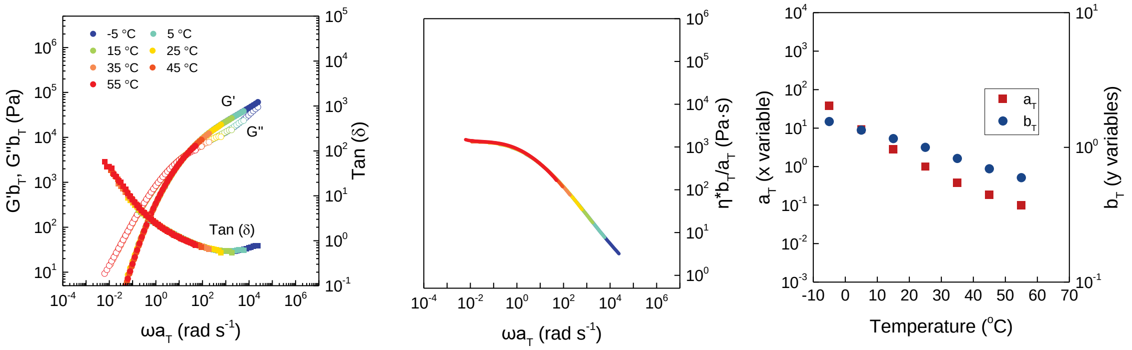

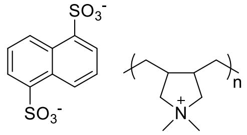

SULF2 PDADMA

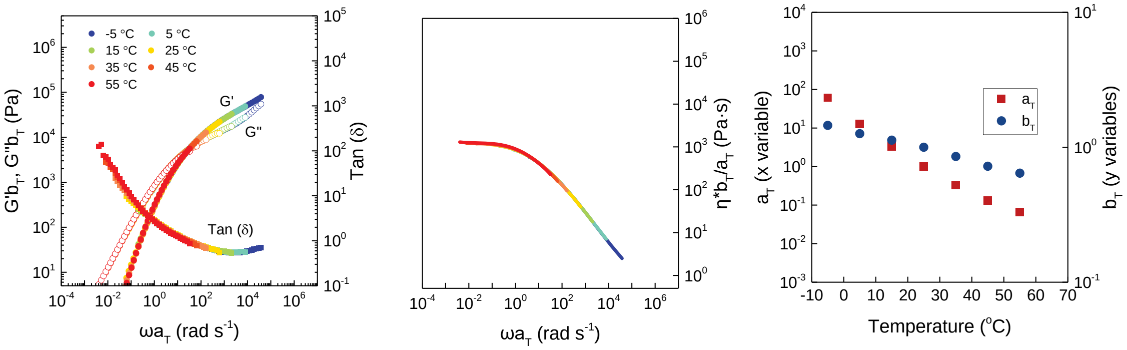

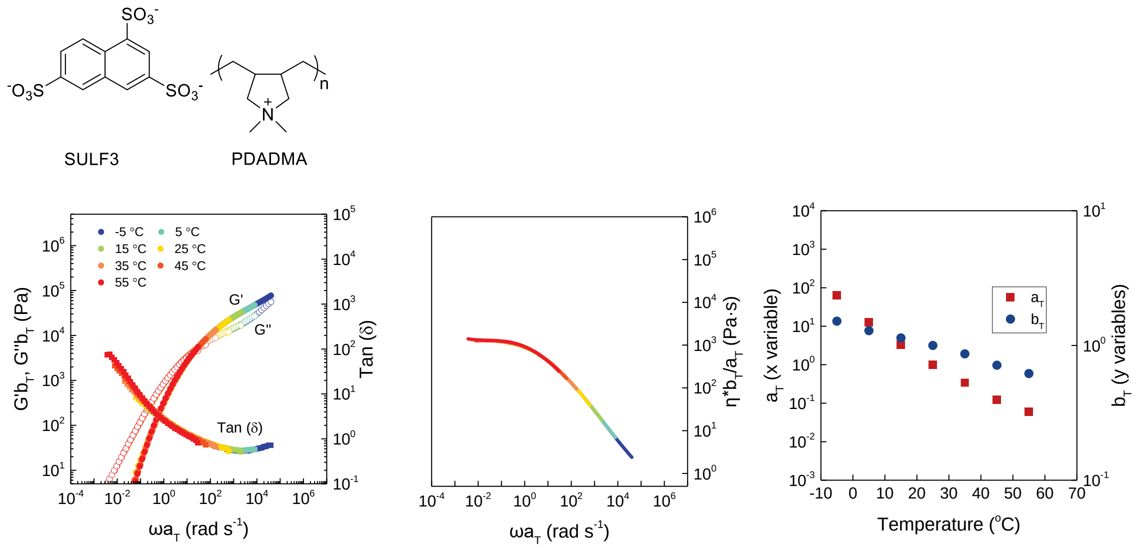

SULF3
PDADMA
-5 °C
5 °C
15 °C
25 °C
35 °C
45 °C
55 °C
G'
G"
Tan (δ)
G'b_T, G"b_T (Pa)
Tan (δ)
ωa_T (rad s^-1)
η*b_T/a_T (Pa·s)
a_T
b_T
a_T (x variable)
b_T (y variables)
Temperature (°C)

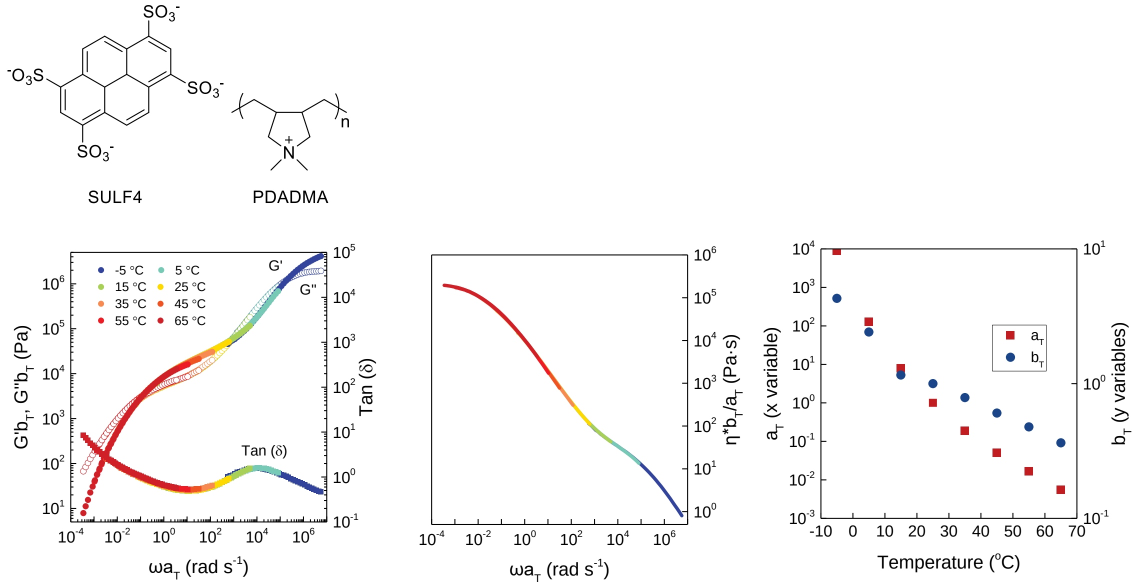

SULF4
PDADMA
-5 °C
5 °C
15 °C
25 °C
35 °C
45 °C
55 °C
65 °C
G'
G"
Tan (δ)
G'b_T, G"b_T (Pa)
Tan (δ)
ωa_T (rad s^-1)
η*b_T/a_T (Pa·s)
a_T
b_T
a_T (x variable)
b_T (y variables)
Temperature (°C)

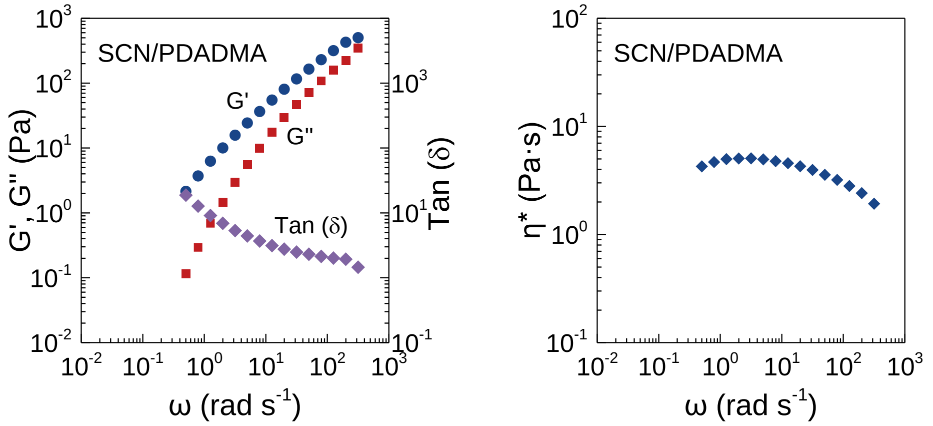

Fig. S3. **Time-temperature superposition of SULF1/PDADMA, SULF2/PDADMA, SULF3/PDADMA, SULF4/PDADMA, and SCN/PDADMA coacervates in 0.01 M NaCl**. G' and G" on the left panels, complex viscosity on the middle panel. Plateau values in viscosity at low frequencies were recorded as zero-shear viscosity. Reference temperature is 25 °C. Frequency responses recorded at different temperatures were shifted along the frequency axis using the shift factor a_T on the right-hand panels.

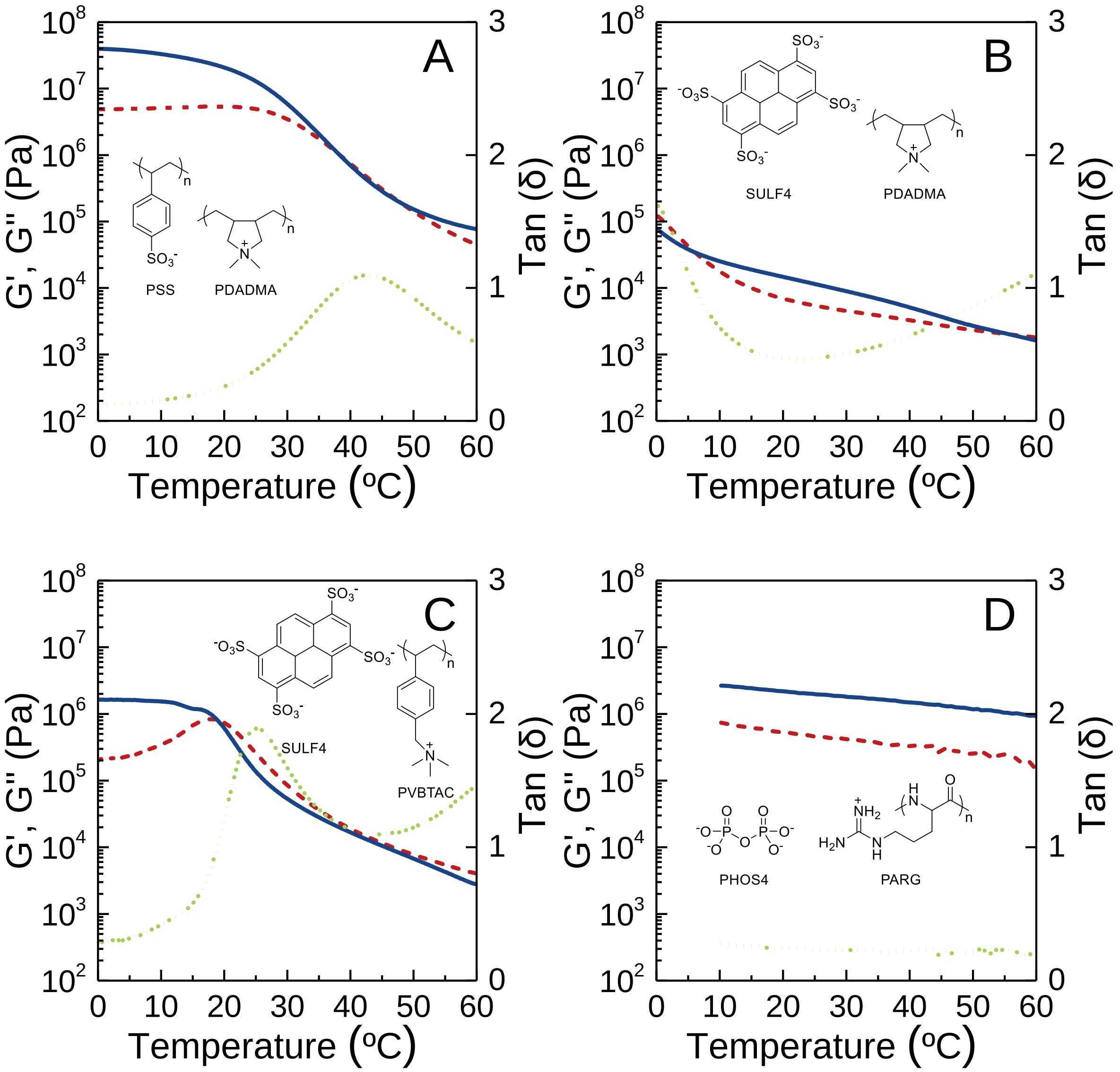

Fig. S4. G’ G” and tan δ versus temperature. A) PSS/PDADMA PEC in 0.01 M NaCl at 1 Hz; B) SULF4/PDADMA in 0.01 M NaCl at 1 Hz; C) SULF4/PVBTA in 0.01 M NaCl at 1 Hz and D) PHOS4/PARG in 0.01 M NaCl at 0.1 Hz. Ramp rate = 1 °C min^{-1} for PSS/PDADMA, SULF4/PDADMA and SULF4/PVBTA, ramp rate = 2 °C min^{-1} for PHOS4/PARG. The peak in tan δ corresponds to a glass transition between glassy (lower temperatures) to rubbery (higher temperatures) material. Solid line, G’; dashed line, G”; dotted line, tan δ.

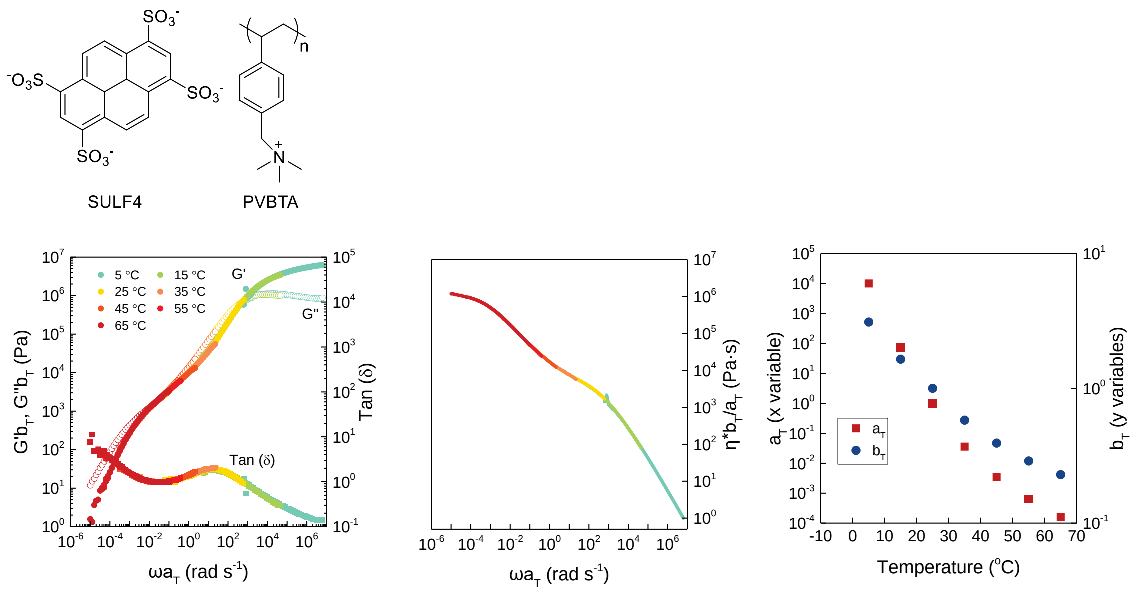

Fig. S5. **Time-temperature superposition of SULF4/PVBTA coacervate (T_g ~ 25 °C) in 0.01 M NaCl.** Reference temperature is 25 °C.

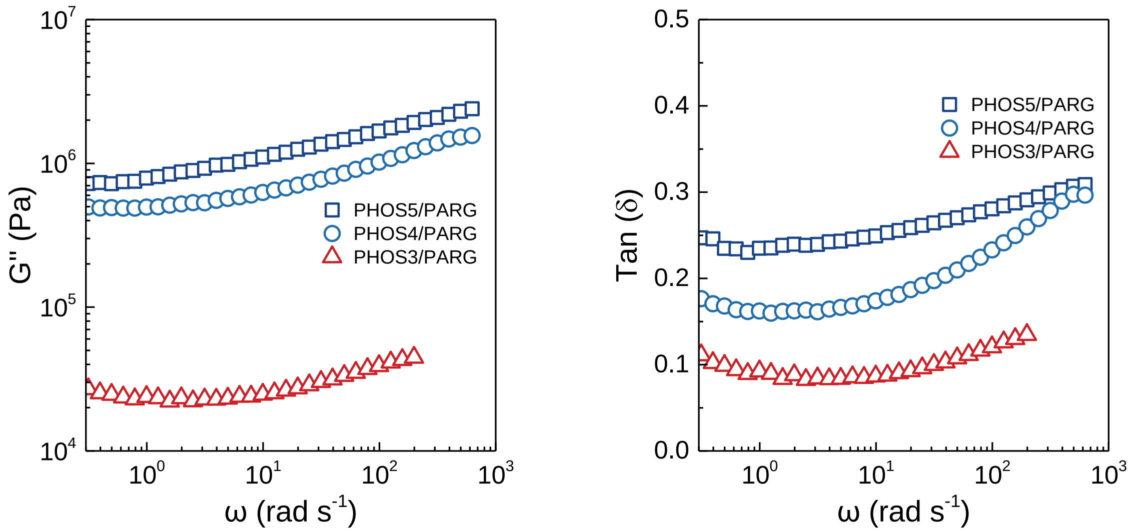

Fig. S6. G'' and tanδ for PHOS/PARG series. At 25 °C.

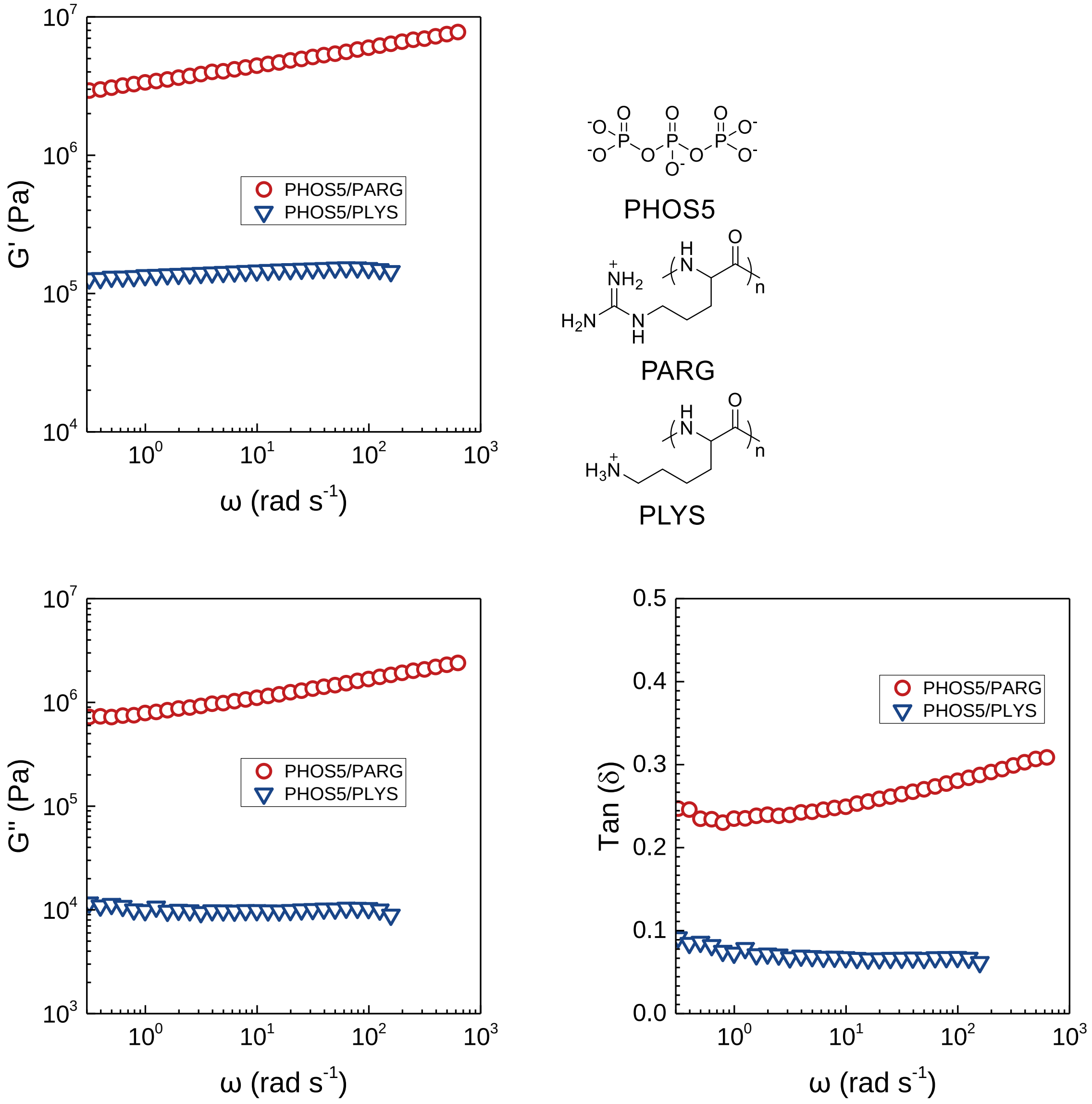

Fig. S7. **G' versus frequency for ○, PHOS5/PARG; ▽, PHOS5/PLYS at 37 °C in 0.15 M NaCl, solution pH = 7**. PARG forms more viscous coacervates.

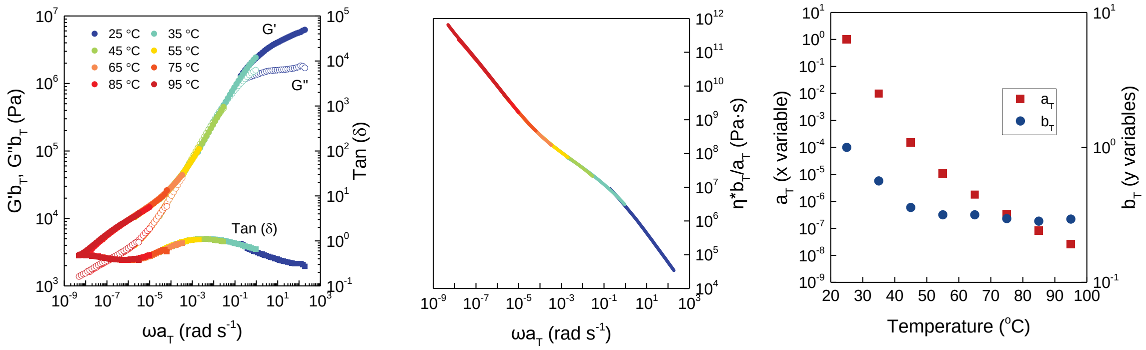

Fig. S8. **Linear viscoelastic response of PSS/PDADMA**. Using time temperature superposition. Reference temperature is 25 °C.

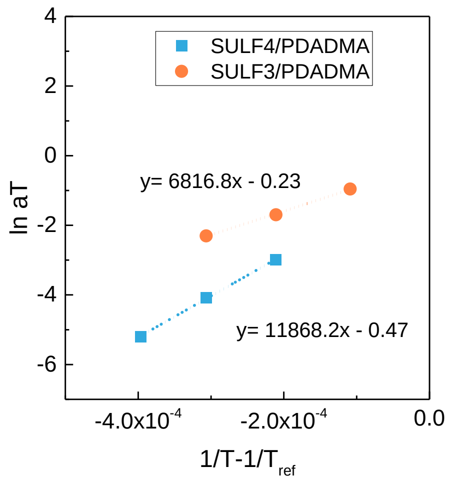

Fig. S9. Arrhenius plot of SULF4/PDADMA and SULF3/PDADMA. Activation energy, E_a, is obtained from the slope of the line, $E_a = 8.314 \times$ slope. For SULF3/PDADMA, $E_a = 56.7$ kJ mol^{-1}, whereas for SULF4/PDADMA, $E_a = 98.7$ kJ mol^{-1}

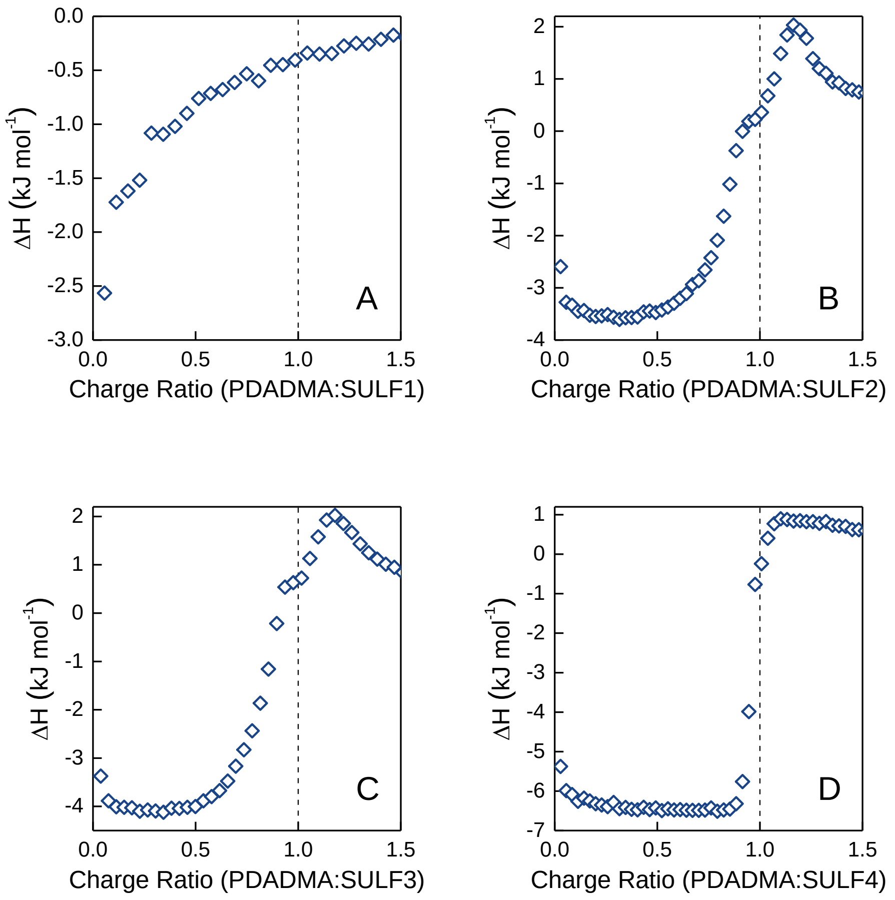

Fig. S10. Isothermal calorimetry titration curves. (A) 10 mM PDADMAC into 0.5 mM SULF1 in 0.05 M NaCl; (B) PDADMAC into 0.5 mM SULF2 in 0.05 M NaCl; (C) 10 mM PDADMAC into 0.25 mM SULF3 in 0.05 M NaCl; (D) 10 mM PDADMAC into 0.25 mM SULF4 in 0.05 M NaCl.
